# Remarks on Medical Education

**Published:** 1852-01

**Authors:** 


### ECLECTIC DEPARTMENT,

### AND SPIRIT OF THE MEDICAL PERIODICAL PRESS

---

### *Remarks on Medical Education*, by Prof. Joseph Roby.

The following remarks on Medical Education are taken from the annual Circular of the Medical Department of the University of Maryland. They are from the pen of Prof. Joseph Roby. As expressing sensible and sound views on a subject in the discussion of which not a little bad feeling and worse logic have been, of late years, expended, they will, we think, commend themselves to the good judgment of the reader.

Editor Buff. Med. Jour.

"Professional education is a department of general mental culture. Its object is to prepare men for a specific use, by instructing them in the knowledge essential for the right fulfillment of such use. It is education preparatory to the actual performance of the duties of a responsible profession; the training of the senses and intelligence to the acquisition of special truths by developing their powers and capacities. No arbitrary formula, no conventional custom, no honorary title can convert the uninitiated into fit followers of a liberal profession, apart from a proper, judicious, well directed system of professional education; a system of careful, continued, methodical, scientific training.

"Such training naturally divides itself into the period immediately antecedent to entrance upon purely professional study, the period more immediately devoted to such study, and the period of active professional life. The preparation for the acquisition of professional knowledge, the acquisition of such knowledge, and the application of it.

"As the object of all education is two-fold, namely, to form certain habits of mind, and to impart a certain amount of useful information, it follows that in preparation for a profession, and especially a profession like medicine, due regard should be paid to each of these particulars; and that the student should be prepared to enter upon scientific study with a fair capacity for using the faculties he may possess, and a proper share of previously acquired knowledge; with habits of mental discipline and with the fruits which such discipline bestows. He should be able so to use his mind as to be qualified, in some measure, to analyze with penetration, to compare with a clear perception of real analogy, to reason with precision, and to apply principles to practice with ready apprehension. He should have acquired, through some process of mental discipline, the ability to understand those truths which may be unfolded to him, and habits of diligent application. He should comprehend clearly that he is not merely to *learn*, but to *know*, and to *know that he knows*. How far such preliminary training should extend, and what it should embrace, may be matters for discussion. The standard will vary with the views of those who may determine it; but that some training is necessary, almost all will concede. Considering the character, position, and prospects of medical science, it can scarcely be regarded, as too exacting or exclusive to insist, that no young man should attempt to enter it who has not acquired, as a sure and indispensable foundation, the elements of a good English education; such as would be requisite for admission to any respectable college. To this it would be well to add some familiarity with natural science, and some knowledge of the ancient languages; the latter, because they are the keys to scientific nomenclature, and because a knowledge of them renders such nomenclature 'pregnant with meaning, which would otherwise be a series of hard, uncouth names.' Above all, the student should be familiar with the structure and use of his own language; be able, literally, to read, write and spell it with accuracy and propriety. Language is the vehicle of thought. No train of reasoning can be followed, with a clear comprehension of the principles involved and the truth it is intended to convey, without ready recognition of the terms in which it is expressed. Such training should be preliminary, because, once entered upon his professional career, the student rarely finds time or inclination for any thing else; and because, through the mental habits it has induced, it will facilitate the acquisition of more purely technical information. The habit of acquiring knowledge cannot be attained at once. It must be formed gradually and continuous; he who has formed it, starts in his course with much advantage. His intellect and his memory will the more readily appropriate and retain the truths which may be offered to him. While it is true, then, that in practicing the art no amount of extra professional learning will aid the physician in the emergencies which he may be called upon to meet; while no degree of scholastic training will stand him in stead in the reduction of a dislocation, the treatment of a hernia, or the management of a fracture, yet the general mental condition resulting from such training will be likely so to influence his habits of thinking and reasoning as to materially aid him in recognizing and recollecting the principles upon which the conduct of the special case may depend.

"This Faculty do not dictate the quantity, or quality, of preliminary education with which the pupil should begin his professional studies. They earnestly urge those who resort to their halls, not to enter upon a study so exacting as the study of medicine must be to those who have a right view of it, with inadequate preparation. The education of the youth generally determines and involves the whole intellectual progress of the man. With rare exceptions, its early defects are seldom remedied, and never but by an amount of self-exertion disproportionate to that needful under a system of judicious timely training.

"The medical schools of this country have been charged with a desire to augment the number, without due regard to the qualifications, of those who enter them. This Faculty disclaim all such desire. They believe that the labors of the teacher are lightened, and his pleasure increased, in direct proportion to the intelligence of those who listen to him. Engaged in teaching a complex science, it would mortify them to feel that their instructions are bestowed upon those who, through lack of previous discipline, original capacity, or general powers of appreciation, are incapable of comprehending and applying them.

"It has been suggested that the school should *require* evidence of preliminary preparation. The requisition would be valuable only so far as it was based upon a careful consideration of the qualifications of those recommended. We are a petition-signing and certificate-giving people: there is scarcely a person or an object for which a voucher cannot readily be obtained. The value of such voucher will depend upon a knowledge of the worthiness of its recipient and of him who gives it: knowledge which it is not always, or even often, easy to procure. It would seem best, therefore, at least for the present, to leave the necessity of adequate preliminary discipline, and the exaction of it, to commend itself, as it will do, to the good sense of the pupil and his instructors—private and public. This Faculty avow their estimate of it, as an intrinsic and indispensable prerequisite, openly and unreservedly; and will always be reluctant to countenance the entrance of any person upon a course of medical study, who has not been subjected to a proper process of preparatory education.

"Granting the utility and need of some previous training as an introduction to the study of medicine, the question arises, how should the study itself be conducted, and with what purpose? The usual plan, in this country, is a combination of private and public instruction. Study under the direction of a private preceptor, and attendance upon the various courses of lectures delivered in medical schools. This system is thought to be a useful and beneficial one, and to place the student in a good position for acquiring a sound professional education. To insure its best effect, private and public teaching must maintain a right relation to each other. Neither of them ought to assume the sole direction; they should be intimately conjoined. A course of systematic private tuition and study is to be regarded as an important part of the process. It should embrace the reading of standard books—not merely those relating to the abstract science, but those which include its history and literature, attention, as far as possible, to its collateral branches, familiar acquisition of its nomenclature, and in relation to its purely demonstrative branches, a fair conception of their nature and value; gradual introduction, under the eye of the instructor, to the application of the knowledge that is acquired, and verification of it by observation and experiment. If the private teacher cannot teach the whole science, he can so train the student as to enable him to perceive clearly what he requires to learn, and assist him in judging how and where the gaps which private study leaves can be best filled up. He can inculcate and enforce the habit of continued, industrious, persevering application; and guide him, as he should always be guided, into a clear perception, that whatever be the amount or variety of knowledge offered to him, it will depend mainly upon himself whether or not it will be rightly appreciated and appropriated. He can test his physical, moral and intellectual fitness for his vocation, and warn him against wasting his time and means in the pursuit of a profession for which he may have no natural adaptation; and he can, and should, do all this, because he stands in more intimate relation to him than the public teacher can possibly attain. This relation, while it helps him to form a correct estimate of the individual character of his pupil, also increases his responsibility to the school and the profession. Whatever theoretical schemes for 'elevating the standard of medical education' may be announced or adopted, the private preceptor should expect to bear his share of the burden. The first step is taken under his care, and often through his advice; it is his duty, therefore, to see that it be not taken hastily or heedlessly. In the tendency which has been manifested to impose the whole responsibility upon the public school, it is to be feared that the position and duties of the private teacher will be overlooked, and that his relation to those whom he professes to instruct, in the sense in which such relation was regarded by the great masters of the art, will be disturbed; that there will be less reverence and love, and less counsel and confidence.

"In conjunction with private study, the student acquires his professional education by resorting to the public school. His object is two-fold; to obtain such knowledge as he cannot so readily get elsewhere, and the credentials which admit him to the ranks of the profession.

"The doctrine that public instruction should cover the whole ground of medical science, is one to which this Faculty do not assent; they doubt, indeed, whether the best digested course of lectures ever did, or ever can, embrace all that is to be learned and taught. They regard lectures as an important and, in some degree, essential means of education, but not as the sole means; and they fear that the teachers who profess to teach, and the pupils who expect to learn, all that is known in the various departments of medical science, even in the most elaborate and extensive course of lectures, will inevitably be disappointed and deluded. They are of opinion, therefore, that public teaching should be directed mainly to those points which cannot be so advantageously attended to in private study; that it should be chiefly demonstrative, and especially that it should have in view, from commencement to conclusion, the preparation of the student for the performance of his practical duties. Whatever may be the case elsewhere, it is unquestionable that, for some years to come, the great body of the American medical profession will be composed of men who cultivate the art more than the science. Their professional life will be actively practical, rather than abstractly scientific; and the great object of education for the mass will be preparation for practice. With us the physician must be an artist, not a philosopher. The fact that whoever would be in, and of, the profession in this country, must be in it as a doer, not merely as a knower, is undeniable. It results from the personal position of most of those who enter it, and from the view taken by the public of their calling and their duties. The aim of a few may be different, but the majority of medical students expect to become practitioners; their final purpose is to become qualified to treat disease. The system of public instruction that does not regard this, cannot be reckoned the most faithful or judicious. Not to teach all that is known—this would scarcely be practicable—but that which is most useful. To bring before the student the subjects which may be chosen, in the most intelligible form; illustrate their relation to his future duties; enforce the principles which govern them; awaken his attention, excite his interest, cultivate his powers of discrimination and observation, stimulate him to the love of knowledge, and develop his capacity for applying it — this is what needs to be done, and what the conscientious teacher will strive to do.

"The branches taught in the schools of this country are, generally, Anatomy and Physiology, Surgery, Theory and Practice, Chemistry, Materia Medica and Obstetrics: all of them important, and all susceptible, in a greater or less degree, of practical illustration and demonstration. To these may be added Medical and Surgical Clinical Instruction and Practical Anatomy. Amidst all the discussions that have arisen in relation to the form, mode, and extent of medical education, two points seem to be universally conceded, viz: that the study of Practical Anatomy and the clinical observation of disease are primitive and essential elements.

"The ground upon which the importance of these branches is based is, the admitted necessity of a knowledge of what they teach as preliminary to entrance upon practice. The physician who knows nothing, from personal examination, of the body with which he deals, is ignorant of the very alphabet of his profession. All his subsequent steps will be hesitating and uncertain, if he have not begun with a well defined outline of structure and function. He cannot follow the details of surgical, medical, or pathological instruction, without some knowledge of the parts to which such instruction constantly refers. True, he may not require to be taught the ultimates of Anatomy, but he must be familiar with its elementary truths. To recognize and reduce a dislocation, he must be acquainted with the form and position of the bones entering into the articulation; to diagnose an aneurism, he must be able to read distinctly the disturbances of natural relation and function. To determine the special characters of a morbid alteration, he must constantly refer to his knowledge of healthy structure.

"The need of accurate anatomical knowledge to the practitioner is universally admitted. It properly belongs to the public school to furnish the facilities for acquiring such knowledge; the time for acquiring it is during the period of professional preparation, and the mode in which it is to be acquired is by diligent personal devotion on the part of the pupil. Deficiencies in other departments of medical education may be made up, to a considerable extent, by subsequent industry and application; but he who fails to become acquainted with the elementary truths of anatomical science during his preparatory pupilage, usually fails forever. Once engaged in the business of his profession, he has no opportunity, time or inclination to submit to the irksome labors of the dissecting room. Whatever else may be taught, or neglected to be taught, attendance upon a course of instruction in Practical Anatomy should be rigidly exacted, and evidence of such attendance should be an essential prerequisite for admission to an examination for graduation.

"A second, and not less important, department of public instruction is included in what are termed 'Clinical Lectures.' By clinical instruction is to be understood, practical observation and illustration of a given case of disease during its whole progress; embracing its diagnosis, prognosis, treatment, and pathology. The mere rehearsal to a medical class, that a 'child has chronic bronchitis;' that this 'generally arises from congestion of the lungs,' which is relieved by secretion, and being 'preceded by measles, there is probably inflammatory action,' and that, 'having been treated with small doses of ipecacuanha, these are to be continued,' with 'a diet of bread and milk, or cream and a little rice flour, the bowels to be kept open with a teaspoonful of castor oil, if necessary,' can hardly be regarded as the highest or most profitable form of clinical instruction; particularly if the child and the class never meet again. True clinical teaching consists in the frequent observation of a disease, with reference to its antecedent history and present condition; the phenomena which develop themselves; the changes which occur, and the effects which remedies produce. If the patient recover, the observation of his convalescence; if he die, the alterations which the disease has induced. A lecture on the general pathology of cancer or tubercle, or the symptomatology of rheumatism or pneumonia, or the signs of dislocations and fractures, aneurism or hernia, is not a *clinical* lecture. It could be given in the public hall quite as well as in the hospital ward. The living illustration must be present, not merely for a transient and casual examination, but for careful and continued observation. Clinical teaching, to answer its best purposes, should address the senses as well as the intellect. It is plain and palpable interpretation of the phenomena of disease. It not only reduces the dislocation, sets the fracture, relieves the hernia, and removes the calculus, but it practically illustrates all the steps of the process; exhibits the difficulties and dangers likely to be encountered, and how to avoid them; and, the operation over, how the subsequent management of the case is to be conducted. In it will be embraced, of course, cases of variable intensity, importance and interest. The chronic case, in which art is apparently inefficient, the incurable, in which she is powerless, and the acute, in which prompt application of her most active agents is essential to the preservation of life. Such teaching it is the object of the school to provide, and attendance upon it should be uniformly required. From clinical instruction alone can the student learn, what he must learn sooner or later, viz, the true relation of Nature and Art. Only in the presence of real disease can he begin to understand the value of a clear conception of the power of remedies, and the rules which should govern their employment. The crude notion that the art which professes to *treat* all disease is able to *cure* all disease, soon vanishes under the lessons of the clinical ward. There he is taught that 'the physician is the minister and interpreter of Nature; let him do or contrive what he will, unless he follows Nature he cannot govern her.'

"To the school the student looks for the credentials which are to admit him to the privileges of his profession. In seeking and bestowing these, the pupil and teacher rest under mutual responsibilities. If made the sole object on the part of either, abuses will inevitably arise. The diligent, intelligent, honorable and upright student, and the faithful, conscientious, discriminating teacher will recognize this responsibility and act under its influence. No man imperfectly prepared for the duties of his profession, has any right to expect that its honors shall be conferred upon him. Yet those who bestow those honors understand the difficulty of their position. They feel that no arbitrary or invariable standard can be established. Amongst a hundred candidates for the Doctorate, it is hardly possible to assume the existence of perfect equality of capacity or acquirement. The elements of a favorable or adverse decision must be based, to some extent, upon a knowledge of average fitness, general character, habits, industry and application. Excellence in one, and that a leading, department, may not unreasonably be allowed to offset moderate deficiency in another and subordinate one. In the most thorough and scientific school in this country, the National Military Academy, this inequality is uniformly recognized. All do not, nor are all expected to, reach the highest standard. The adage, '*Nihil simul inventum est et*

*perfectum,'* is as applicable in medicine as in law.* In admitting the candidate to the honors he desires to obtain, it would seem best that the test be a discriminating one, and that he be dealt with justly and liberally. By 'liberally' is not meant, that 'the requirements for admission into the profession should be placed so low that the door may be open to all, with as little expenditure as possible of time, money and industry, and with proportionably as small an amount of qualification.' This Faculty have neither collectively nor individually entertained or expressed such opinions. They simply mean that all the elements of the candidate's moral and intellectual character should be allowed due weight, and that his acceptance or rejection should be founded, if possible, upon actual acquaintance obtained through previous intercourse. The test of a single hour's examination can go but little way toward forming a strictly just estimate of capacity and knowledge. The former may pass for more than its value, and the latter may be merely apparent, not real. It is in this respect that frequent oral examinations are of use; they enable the teacher to calculate the intellectual ability of his pupil. They are also beneficial as *means* of education. The habit of submitting to them induces readiness, thoughtfulness, and self-possession; trains the student to express himself with precision, tests his comprehension and recollection of the truths taught, insures greater punctuality of attendance, closer attention, excites emulation, and renders the duty of a final decision less perplexing and laborious.

"Lastly, it should be the object of professional education to inspire such love for knowledge, and excite such desire for truth as shall create permanent habits of acquisition and improvement. Education consists not merely in imparting information, but in educing the faculties of man. It is literally the formation of the mind. It must be progressive; and its best purpose will remain unfulfilled, if the student carry with him into the scenes of active life, the delusion that the degree of culture he has received, through private or public teaching, is to be final and complete. Once recognized as a legitimate follower of a liberal science, it becomes him to remember that it is a progressive science, and that no one can make good his position in it who does not keep pace with its progress. As its highest object is truth, and its best use the doing of good, his intellect must be active to discover truth and develop good."

---

### *Epidemic Dysentery among the Orphans at Girard College.*

Dr. Hollingsworth stated that a severe epidemic of diarrhœa and dysentery had recently prevailed among the orphans at the Girard College. It commenced on the fourth day of last month, since which time upward of one hundred cases had occurred; namely, twenty-five of dysentery, and the

---

* It has been suggested that there should be two grades in Medicine—"Bachelor of Physic" and "Doctor." This is the plan of the English Universities. The University of Maryland confers both degrees, and, for a time, this was the custom at Harvard University; the degree of "Doctor" being given after a period of years had been passed as "Bachelor." The practical result was, that the "Bachelor," finding himself popularly recognized and regarded as "Doctor," seldom took pains to obtain a second degree, and the custom was abandoned.

